# Histomorphological studies of broiler chicken fed diets supplemented with either raw or enzyme treated dandelion leaves and fenugreek seeds

**DOI:** 10.14202/vetworld.2016.269-275

**Published:** 2016-03-12

**Authors:** Saim Qureshi, Mohammed Tufail Banday, Irfan Shakeel, Sheikh Adil, Masood Saleem Mir, Yasir Afzal Beigh, Umar Amin

**Affiliations:** 1 Division of Livestock Production and Management, Faculty of Veterinary Sciences and Animal Husbandry, Sher-e-Kashmir University of Agricultural Sciences & Technology of Kashmir, Shuhama - 190 006, Srinagar, Jammu and Kashmir, India; 2 Division of Veterinary Pathology, Faculty of Veterinary Sciences and Animal Husbandry, Sher-e-Kashmir University of Agricultural Sciences & Technology of Kashmir, Shuhama - 190 006, Srinagar, Jammu and Kashmir, India; 3 Division of Animal Nutrition, Faculty of Veterinary Sciences and Animal Husbandry, Sher-e-Kashmir University of Agricultural Sciences & Technology of Kashmir, Shuhama -190 006, Srinagar, Jammu and Kashmir, India

**Keywords:** broiler chicken, dandelion, fenugreek, histomorphology

## Abstract

**Aim:**

Herbal plants and their derived products are extensively used particularly in many Asian, African, and other countries of the world as they are considered as ideal feed additives because of their non-residual effect and ability to influence the ecosystem of gastrointestinal microbiota in a positive way. Further, the enzymatic treatment of these herbs helps in their efficient utilization by the host. Dandelion leaves and fenugreek seeds have been reported to have positive effect in terms of improving the performance of broiler chicken, but not much literature is available regarding their effect on gut histomorphology; therefore, the present study was conducted to explore the effect of these herbs either alone or in combination with or without enzyme treatment on histomorphology of liver and small intestine of broiler chicken.

**Materials and Methods:**

To achieve the envisaged objective, 273-day-old commercial broiler chicks were procured from a reputed source and reared together until 7 days of age. On the 7^th^ day, the chicks were individually weighed, distributed randomly into 7 groups of 3 replicates with 13 chicks each. Birds in the control group were fed diets without additives (T_1_). The other six treatment groups were fed the basal diet supplemented with 0.5% dandelion leaves (T_2_), 1% fenugreek seeds (T_3_), combination of 0.5% dandelion leaves and 1% fenugreek seeds (T_4_), enzyme treated dandelion leaves 0.5% (T_5_), enzyme treated fenugreek seeds 1% (T_6_), and combination of enzyme treated dandelion leaves (0.5%) and (1%) fenugreek seeds (T_7_). The histomorphological study of liver and small intestines was conducted among different treatment groups.

**Results:**

The results revealed the hepato-protective nature of both dandelion leaves and fenugreek seeds either alone or in combination with or without enzyme treatment when compared with the control group. Moreover, the histomorphological findings of jejunum revealed the beneficial effect of dandelion leaves, fenugreek seeds and enzymes on the intestinal mucosa in terms of cellular infiltration, architecture of villi, villus height/crypt depth ratio, thereby improving the intestinal health.

**Conclusion:**

The dandelion leaves and fenugreek seeds have hepato-protective nature and beneficial effect on the intestinal morphology particularly when included along with enzymes in the diet of broiler chicken.

## Introduction

Phytogenics are a group of natural growth promoters or non-antibiotic growth promoters derived from herbs, spices, or other plants. Compared with synthetic antibiotics or inorganic chemicals, these plant-derived products have proven to be safe, less toxic, residue free and are thought to be ideal feed additives in food animal production. Phytogenic feed additives have gained increasing interest, especially for their application in poultry diets [1]. They beneficially affect the ecosystem of gastrointestinal microbiota through controlling potential pathogens and improving digestive capacity in the small intestine and stabilizing the microbial eubiosis in the gut [2]. The herbal plant extracts help in the alteration of intestinal microbiota, increase of enzyme secretion, improvement of the immune response, and histomorphological maintenance of the gastrointestinal tract [3].

Kashmir, often referred to as paradise on earth, is located at the northwestern tip of Himalayan biodiversity hotspot [4]. The region supports a rich and spectacular plant biodiversity of great scientific curiosity and promising economic benefits. Among the herbal flora available in the region, two herbal plants, i.e., dandelion leaves (*Taraxacum officinale*) and seeds of fenugreek (*Trigonella foenum-graecum*) were utilized for the study of the diets of the broiler chicken. Dandelion is a well-known medicinal plant that grows in nature in Asia, Europe, and North America [5]. The roots of the herb are primarily considered for supporting digestion and liver function, while as its leaves are used as diuretic and digestive stimulant [6]. Fenugreek is grown mainly in India, Pakistan, and China. Its seeds have many therapeutical effects such as hypoglycemic, anti-helminthic, anti-inflammatory, and anti-microbial properties [7]. It also contains lecithin and choline that help to dissolve cholesterol and fatty substances. It also contains neurin, biotin, and trimethylamine which tends to stimulate the appetite by their action on the nervous system [8]. The dietary supplementation of dandelion leaves and fenugreek seeds have been reported to increase the performance of broiler chicken [9-11]. Enzyme supplementation in poultry diets has been reported to improve the performance [12] by degrading non-starchy polysaccharides, improving the digestion and absorption of nutrients [13], and improving their intestinal morphology [14].

Since, dandelion leaves and fenugreek seeds were reported to have positive effect in terms of improving the performance of broiler chicken, but not much literature is available regarding their effect on gut histomorphology; therefore, the present study was conducted to explore the effect of these herbs either alone or in combination with or without enzyme treatment on histomorphology of liver and small intestine of broiler chicken.

## Materials and Methods

### Ethical approval

The study was conducted after approval of research committee and institutional ethical committee.

### Methodology

273-day-old commercial broiler chicks procured from a reputed source were utilized for the study which lasted 42 days. Chicks were reared in battery cages until 7 days of age. During this period, all the birds were provided with a pre-starter mash (23% crude protein and 2800 Kcal/kg metabolizable energy). Birds had free access to feed and water throughout and were maintained on a constant 24 h light schedule. On the 8^th^ day, the chicks were individually weighed, distributed into seven treatment groups of three replicates with 13 chicks in each in a completely randomized design so that the treatment means differ as little as possible. Birds in the control group were fed diets without additives (T_1_). The other six treatment groups were fed the basal diet supplemented with 0.5% dandelion leaves (T_2_), 1% fenugreek seeds (T_3_), combination of 0.5% dandelion leaves and 1% fenugreek seeds (T_4_), enzyme treated dandelion leaves 0.5% (T_5_), enzyme treated fenugreek seeds 1% (T_6_), and combination of enzyme treated dandelion leaves (0.5%) and (1%) fenugreek seeds (T_7_). The diets were formulated to meet the recommendations of the Bureau of Indian standards [15]. Dandelion leaves (*T. officinale*) and fenugreek seeds (*T. foenum-graecum*) were procured, dried and in powder form mixed thoroughly in aforesaid quantities to a small amount of feed (1 kg) in a premixer. The resultant mixture was then mixed with the rest of the feed in a mechanical blender until a thorough and consistent mixture was obtained. All chicks were vaccinated against Ranikhet disease on the 5th day with F1 strain vaccine and infectious bronchitis virus-95 vaccine against infectious bursal disease on the 16th day. Chicks were checked twice daily for mortality if any. Birds were kept under the same managerial, hygienic, and environmental conditions.

### Parameters recorded

For the histopathological analysis, the tissue samples from liver (main organ of biotransformation) jejunum (main site for absorption) were collected from the slaughtered birds (6 birds per treatment) at the end of experimental period (42 days) and fixed in 10% buffered formalin saline. Tissues were dehydrated by immersing through a series of alcohols of increasing concentrations (from 70% to absolute), infiltrated with xylene, and embedded in paraffin. The casting of blocks was carried out in L-molds (two L-shaped pieces) which facilitated the manipulation of size as per the requirement. The rotary type microtome was used for cutting the paraffin sections. The blocks were properly trimmed, and the sections of 5 mm thickness were cut. Continuous ribbons (6-7 inches long) of the material were cut and laid on the surface of constant temperature water bath (around 55°C). The sections were separated with a heated scalpel after they spread completely. The cut sections were mounted on the clean glass slides using Mayer’s egg albumin as the section adhesive. The mounted slides were dried in paraffin oven at 60°C for 1 h. The tissue sections were stained by the Harris hematoxylin and eosin staining method. The paraffin sections were deparaffinized with the xylene before hydration through graded alcohol to distilled water. This was followed by the dehydration in ascending grades of alcohol. The clearing was performed in the xylene, and a drop of distrene plasticizer xylene mountant was placed on a coverslip and the section on the slide pressed on it. The slide was inverted, and the cover slip was pressed with a rod to remove the air bubbles if any trapped. The values were measured with an oculometer at a magnification of ×10 under a light microscope fitted with the stage micrometer.

## Results

### Liver

**Figure-1:**
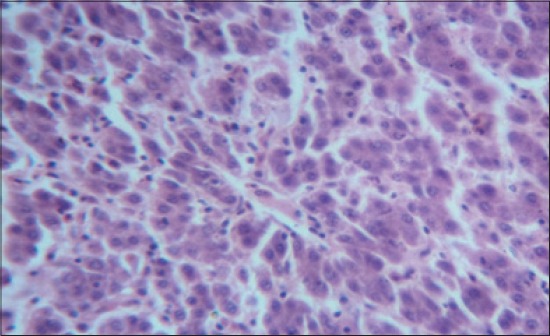
Liver from control group (T_1_) showing mild degree of inflammation with occasional Kupffer cell hyperplasia and distortion of hepatic cords (H and E, ×4).

**Figure-2:**
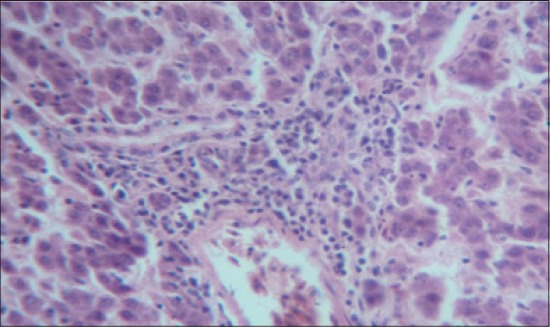
Liver from control group (T_1_) showing heterophil aggregates (H and E, ×4).

**Figure-3:**
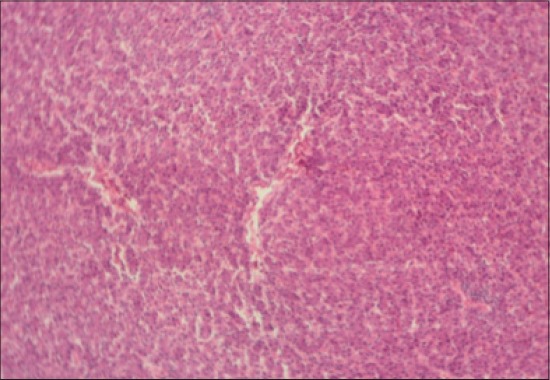
Liver from T_2_ showing occasional and only mild perivascular mononuclear cell infiltration (H and E, ×4).

**Figure-4:**
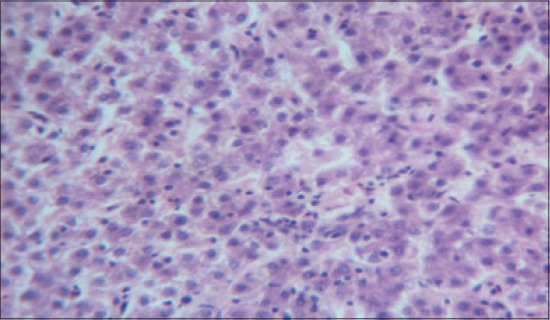
Liver from T_3_ showing less Kupffer cell hyperplasia and perivascular mononuclear cell infiltration (H and E, ×4).

**Figure-5:**
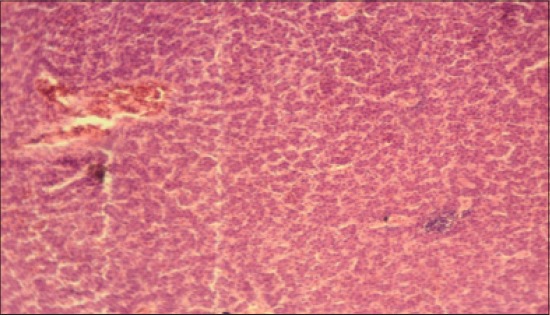
Liver from T_4_ showing hepatocyte regeneration and less heterophil infiltration (H and E, ×4).

**Figure-6:**
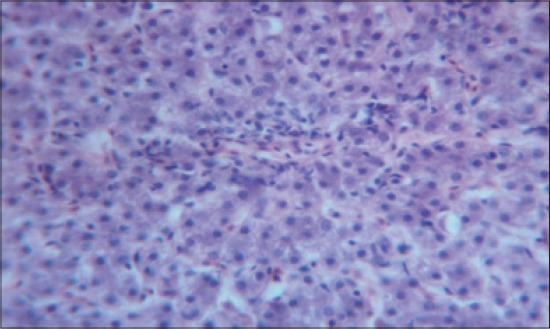
Liver from T_5_ showing less hepatocellular degenerative changes and diffuse inflammatory cell infiltration (H and E, ×4).

**Figure-7:**
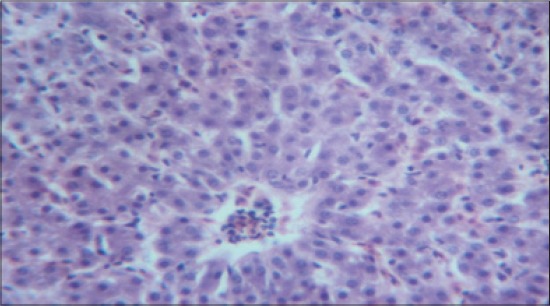
Liver from T_6_ showing less hepatocellular degenerative changes and diffuse inflammatory cell infiltration (H and E, ×4).

**Figure-8:**
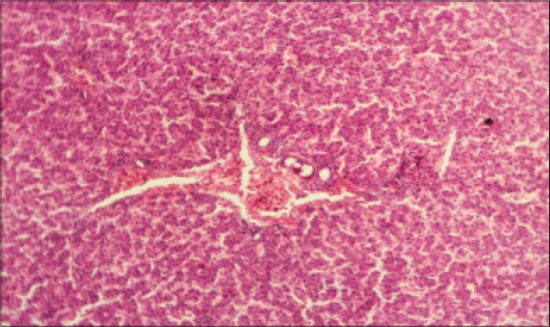
Liver from T_7_ showing normal architecture and beneficial effect on liver (H and E, ×4).

The histomorphological examination of liver from the control group (T_1_) showed a mild degree of hepatocellular degeneration with occasional distortion of hepatic cords associated with Kupffer cell hyperplasia. Frequently marked mononuclear cell infiltration was observed in perivascular area and occasional heterophil forming aggregates of various sizes were noted in the liver parenchyma (Figures-1 and 2). Compared to control, T_2_ group had occasional and only mild perivascular mononuclear cell infiltration (Figure-3). The T_3_ group showed less hepatocellular degeneration, Kupffer cell hyperplasia and perivascular mononuclear cell infiltration and occasional heterophil aggravate in the parenchyma, but the changes were less severe than the control group (Figure-4). Liver from T_4_ showed hepatocyte regeneration (Figure-5), T_5_ (Figure-6), and T_6_ (Figure-7) had less hepatocellular degenerative changes and diffuse inflammatory cell infiltration. The Group T_7_ showed normal architecture and beneficial effect on liver (Figure-8).

### Small intestine

**Figure-9:**
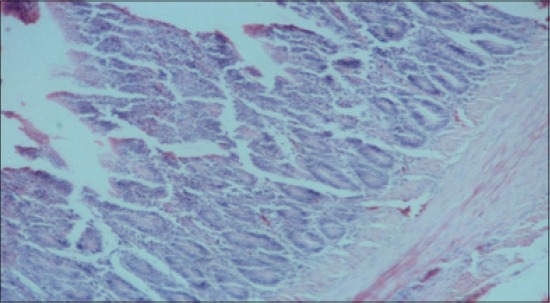
Jejunum from control group (T_1_) showing mucous degeneration of glandular epithelium at certain places (H and E, ×2.5).

**Figure-10:**
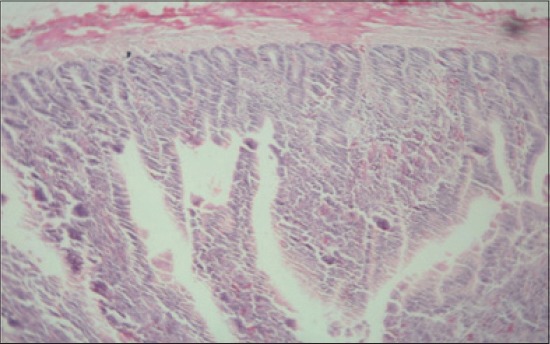
Jejunum from control group (T_1_) showing diffuse infiltration of inflammatory cells, predominantly heterophils and clubbing of villi (H and E, ×2.5).

**Figure-11:**
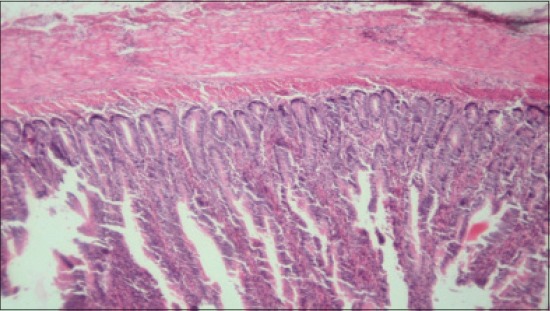
Jejunum from T_2_ showing normal villus architecture with mild cellular infiltration in mucosa and sub-mucosa (H and E, ×2.5).

**Figure-12:**
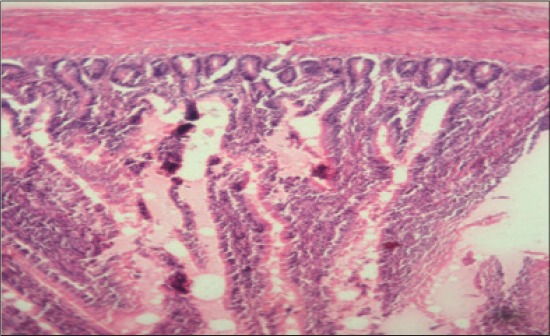
Jejunum from T_3_ showing normal villus architecture with increase in thickness of villus, less and occasional areas of distortion (H and E, ×2.5).

**Figure-13:**
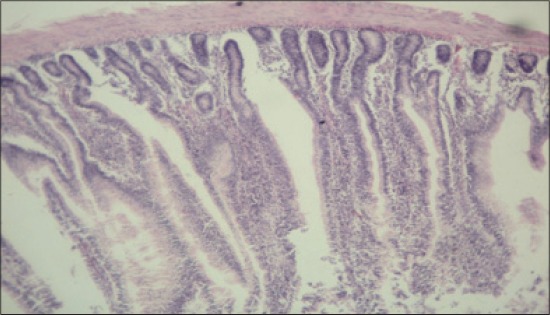
Jejunum from T_4_ showing normal villus architecture, increase in height of villi (H and E, ×2.5).

**Figure-14:**
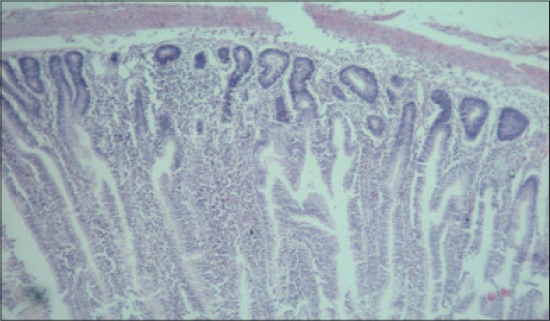
Jejunum from T_5_ showing normal villus architecture, increase in height of villus, occasional areas of cellular infiltration (H and E, ×2.5).

**Figure-15:**
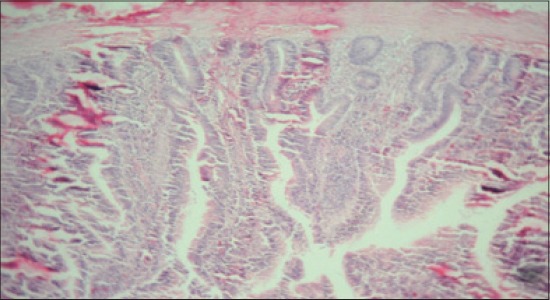
Jejunum from T_6_ showing thickening of villi and increase in height of villi (H and E, ×2.5).

**Figure-16:**
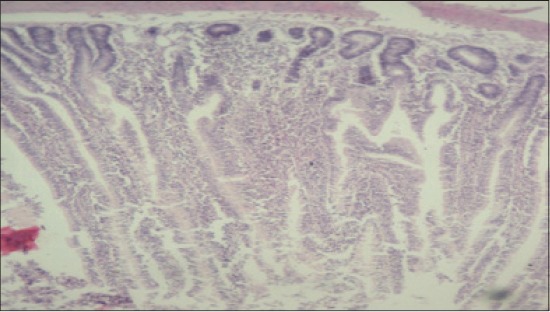
Jejunum from T_7_ showing normal villus architecture with increase in thickness and height of villus, occasional areas of cellular infiltration compared to control group (H and E, ×2.5).

The histological examination of jejunum showed an improvement in the jejunal villus height (p<0.05) and villus height/crypt depth ratio in all the groups fed dandelion leaves and fenugreek seeds with or without enzyme treatment compared to control (Table-1). The control group (T_1_) showed mucous degeneration of glandular epithelium at certain places, diffuse infiltration of inflammatory cells, predominantly heterophils in mucosa in addition to clubbing of villi (Figures-9 and 10). The T_2_ group had normal villus architecture with mild cellular infiltration in mucosa and sub-mucosa; occasional clubbing of villi with less severe infiltration of inflammatory cells than control group was noted (Figure-11). The Group T3 revealed an increase in thickness and height of villi as compared to control and mild cellular infiltration with occasional distortion of villi was also observed (Figure-12). Compared to control, T_4_ showed normal villus architecture and increase in height of villi (Figure-13). Jejunum from T_5_, T_6_, and T_7_ had normal villus architecture, increase in height of villus and occasional areas of cellular infiltration than T_1_ (Figures-14-16).

**Table-1:** Effect of dandelion leaves and fenugreek seeds on jejunal histomorphology of broiler chicken.

Parameter	Treatments						

	T_1_	T_2_	T_3_	T_4_	T_5_	T_6_	T_7_
Villus height (µm)	995.7±14.55^a^	1019.4±10.24^ab^	1032.3±11.54^abc^	1064.2±12.53^cd^	1042.3±8.48^bc^	1038.0±14.87^bc^	1088.3±5.93^d^
Crypt depth (µm)	158.3±3.27	156.4.1±1.54	157.7±5.32	152.4±5.68	155.6±4.97	152.4.±5.91	151.1±3.05
Villus height/crypt depth ratio	6.29±0.07	6.51±0.10	6.55±0.15	7.01±0.35	6.71±0.24	6.83±0.32	7.20±0.13

Means within the same row with different superscripts are significantly different (p<0.05)

## Discussion

### Liver

The histological examination of liver in different treatment groups revealed the hepato-protective role of both dandelion leaves and fenugreek seeds. There was increased hepato-regeneration in the treatment groups where dandelion leaves and fenugreek seeds were supplemented in the diet when compared to the control group. Further, there was a decrease in Kupffer cell hyperplasia and perivascular mononuclear cell infiltration. Best results were observed where both dandelion leaves and fenugreek seeds were used along with enzymes (T_7_). The results of the present study coincide with the results of Park *et al*. [16], Tabassum *et al*. [17], Al-Malki *et al*. [18], and Gulfraz *et al*. [19] who reported the hepato-protective role of dandelion leaves. Likewise, fenugreek seeds have also been reported to have a hepato-protective impact [20,21]. The hepato-protective role of dandelion leaves might be attributed to the bioactive components present in it such as Vitamins (A, C, thiamine, and riboflavin), sesquiterpene lactones, triterpenes, carotenoids (lutein), fatty acids (myristic), and flavonoids (apigenin and luteolin) present in it [17,22,23]. Bitter compounds present in dandelion leaves have been reported to increase the production of bile from gall bladder thereby improving the liver function [24]. Further, the hepato-protective role of fenugreek seeds might be attributed to the bioactive ingredients present in it, which enhance hepatic function and due to its antioxidant activity as reported by Bukhari *et al.*, [25] who reported the antioxidant capacity of the fenugreek extracts.

### Small intestine

The histological examination of jejunum revealed an improvement in villus height, villus height/crypt depth ratio, and less mononuclear cellular infiltration in all the groups fed either dandelion leaves or fenugreek seeds alone or in combination with or without enzyme treatment when compared with the control group. Best results were obtained in the treatment group where the combination of enzyme treated dandelion leaves and fenugreek seeds were used. Increased villus height helps to enhance the absorptive surface area for better utilization of nutrients as reported by Adil *et al*. [26] with the use of feed additives in broiler chicken. Similarly, Abdel-Rahman *et al*. [27] and Debnath *et al*. [28] reported that the supplementation of herbal products in the diet of broiler chicken enhance intestinal villus height and surface area, resulting in better intestinal health. The short or damaged villi impair the absorption of the intestine, which might lead to poor performance of birds [29]. In addition, enzyme supplementation has been reported to improve villus height and villus height/crypt depth ratio in poultry [14,30]. Moreover, in the present study, the jejunal crypt depth decreased non-significantly in the treatment groups compared to control. The crypts are responsible for production of enterocytes required for renewal of villi and the more the crypt is demanded in terms of cell renewal, the greater its depth [31], thus indicating that the villi were not compromised in any way in all the treatment groups fed dandelion leaves and fenugreek seeds either alone or in combination with or without enzyme addition. Furthermore, there was an improvement in the villus height/crypt depth ratio in all the treatment groups which has been regarded as a good indicator of better intestinal health [31]. The beneficial effect on jejunal histomorphology by dandelion leaves and fenugreek seeds might be attributed to their anti-microbial action which in turn has been reported to decreases the inflammatory reactions at the mucosa, thereby increasing the villus height [32,33]. Additional improvement in the intestinal morphology by the addition of enzymes may be because dietary inclusion of the enzymes helps to degrade the non-starch polysaccharides and diminish their negative impact on the gut morphology as they have been reported to suppress the gut morphological development at higher levels [14].

## Conclusion

The dandelion leaves and fenugreek seeds have hepato-protective nature and beneficial effect on the intestinal morphology, particularly when included along with enzymes in the diet of broiler chicken.

## Authors’ Contributions

This study is the part of M.V.Sc. Thesis of the first author SQ, who carried out the research under the guidance of MTB. IS, UA helped during the trial and MSM in the processing of samples. SA provided necessary guidelines during the work and helped in the technical writing of the article. YAB helped in thorough revision of the manuscript. All authors have read and approved the final version of the manuscript.
